# The long non-coding RNA SNHG14 inhibits cell proliferation and invasion and promotes apoptosis by sponging miR-92a-3p in glioma

**DOI:** 10.18632/oncotarget.23960

**Published:** 2018-01-04

**Authors:** Qiang Wang, Yiwan Teng, Rong Wang, Danni Deng, Yijie You, Ya Peng, Naiyuan Shao, Feng Zhi

**Affiliations:** ^1^ Department of Neurosurgery, The First People’s Hospital of Changzhou, Changzhou, Jiangsu, China; ^2^ Changzhou Center for Biotech Development, Changzhou, Jiangsu, China; ^3^ Modern Medical Research Center, The Third Affiliated Hospital of Soochow University, Changzhou, Jiangsu, China

**Keywords:** SNHG14, miR-92a-3p, glioma, prolifertion, invasion

## Abstract

Malignant glioma is one of the most common types of primary brain tumours. Long non-coding RNAs (lncRNAs) have recently emerged as a new class of therapeutic targets for many cancers. In this study, we aimed to explore the functional involvement of small nucleolar RNA host gene 14 (SNHG14) and its potential regulatory mechanism in glioma progression. SNHG14 was found to be downregulated in human glioma tissues and cell lines. SNHG14 significantly inhibited cell viability, reduced cell invasion, and induced apoptosis in glioma cell lines. Furthermore, a correlation analysis demonstrated that there was a negative correlation between SNHG14 expression and miR-92a-3p expression. Bioinformatics prediction and luciferase reporter assays demonstrated that miR-92a-3p could directly bind to SNHG14. miR-92a-3p was significantly upregulated in glioma and acted as an oncogene in glioma cells by inhibiting Bim. Moreover, mechanistic investigations showed that miR-92a-3p could reverse the tumour suppressive effects induced by SNHG14 in glioma, indicating that SNHG14 may act as an endogenous sponge that competes for binding to miR-92a-3p. Our results suggest that SNHG14 and miR-92a-3p may be promising molecular targets for glioma therapy.

## INTRODUCTION

Glioma is one of the most common brain tumours, accounting for 70% of adult malignant primary brain tumours [1]. Glioblastoma (GBM), which is the most aggressive glioma, has a poor prognosis and a high mortality rate in its advanced stage [2]. Although the diagnosis and treatment strategies for glioma have been greatly improved, the prognosis for glioma is still poor due to its malignant proliferation and invasion [3]. Therefore, it is necessary to identify novel therapeutic targets and elucidate new molecular mechanisms for the treatment of glioma.

Long non-coding RNAs (lncRNAs) are transcripts that are longer than 200 nucleotides without protein-coding potential [4]. LncRNAs are implicated in a wide range of cellular processes, including cell proliferation, invasion, apoptosis, and chemoresistance [5–7]. Furthermore, many studies have shown that lncRNAs can serve as oncogenes or tumour suppressors through their interactions with tumour-related genes and signalling pathways in tumourigenesis [8, 9]. Small nucleolar RNA host gene 14 (SNHG14) is located within the Prader-Willi critical region and extends in antisense into the region of the ubiquitin protein ligase E3A (UBE3A) gene, whose deficiency in brain cells in children causes neurogenetic disorders, such as Angelman syndrome [10, 11]. It has been reported that SNHG14 exacerbates cerebral infarction through microglia activation due to miR-145-5p inhibition [11]; however, there is limited research on the relationship between SNHG14 expression and glioma progression, and the underlying mechanism of SNHG14 remains poorly understood in glioma.

MicroRNAs (miRNAs) are small non-coding RNA molecules (18-25 nucleotides) that act as endogenous suppressors of gene expression to induce mRNA cleavage or translational repression, mainly by binding to the 3’-untranslated region (3’-UTR) of their target mRNAs [12, 13]. Since their discovery, miRNAs have been extensively reported to be involved in the development of various cancers, including glioma [14]. Recent studies have shown that lncRNAs have intrinsic miRNA sponging properties that regulate the target genes of miRNAs [15].

In the present study, we aimed to discover the underlying molecular mechanism of SNHG14 in glioma progression. First, we measured the expression levels of SNHG14 in glioma tissues and cell lines and found its downregulation in glioma. Next, we identified the tumour suppressive role of SNHG14 in glioma progression based on functional experiments. Then, we found that there was a negative correlation between SNHG14 and miR-92a-3p. Additionally, miR-92a-3p could directly bind to SNHG14. Furthermore, we found that miR-92a-3p acted as an oncogene in glioma. Finally, mechanistic investigations revealed that SNHG14 inhibited glioma progression by acting as a sponge for miR-92a-3p. Taken together, these results suggest that SNHG14 and miR-92a-3p may be potential therapeutic targets for glioma therapy.

## RESULTS

### SNHG14 is downregulated in human glioma tissues and cell lines

**Figure 1:**
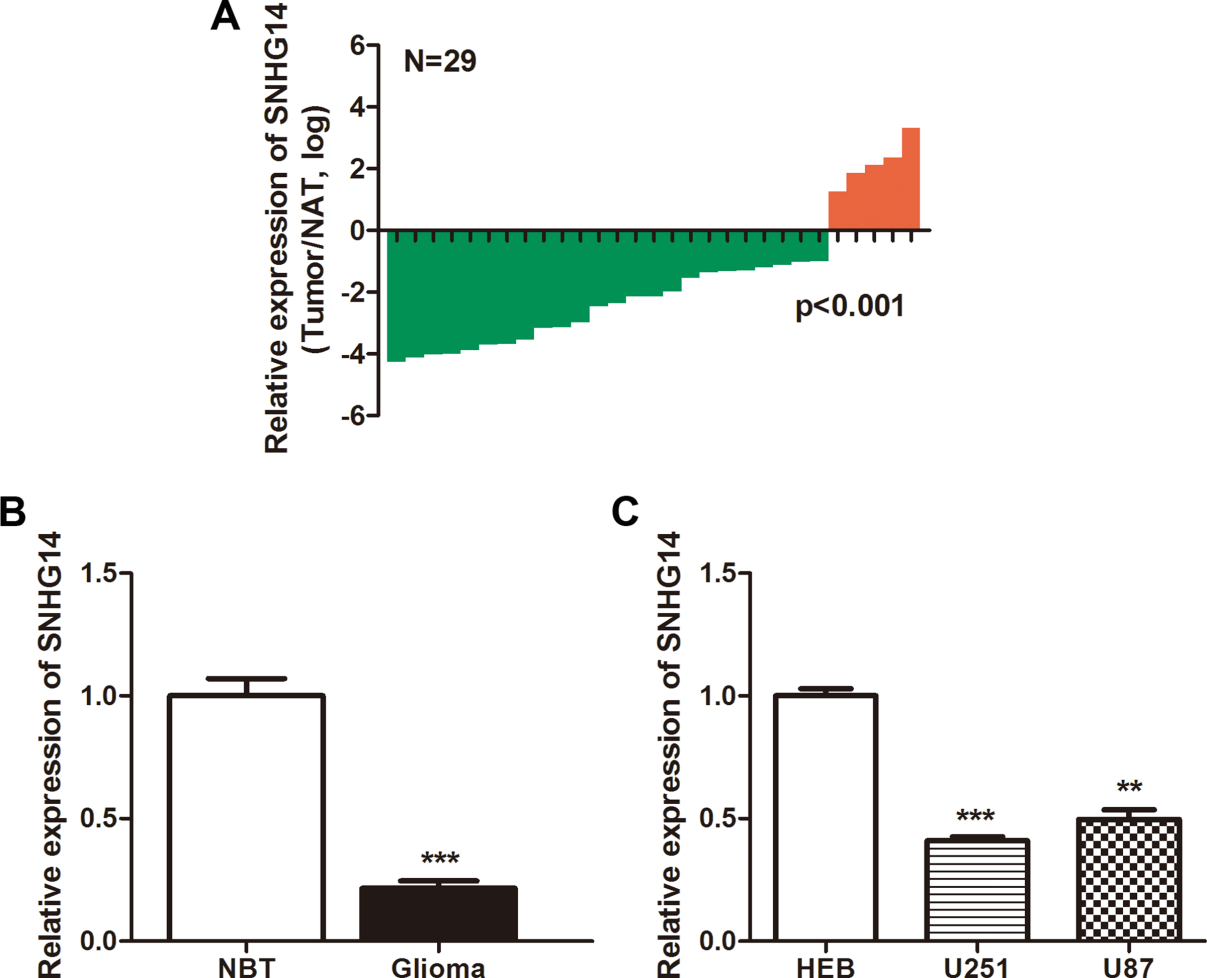
LncRNA SNHG14 expression in human glioma tissues and cell lines (**A**) SNHG14 was significantly downregulated in human glioma tissues. The green shading indicates the downregulation of SNHG14, and the data are presented as the fold-change in tumour tissues relative to NATs assessed by qRT-PCR. *p* < 0.001. (**B**) SNHG14 expression in glioma tissues was significantly lower than that in NBTs. ^***^p < 0.001. (**C**) SNHG14 expression in glioma cell lines (U251 and U87) was significantly lower than that in the HEB cell line. ^**^p < 0.01, ^***^p < 0.001.

The relative expression levels of SNHG14 were measured in 29 paired glioma tissues and their corresponding NATs using qRT-PCR and were normalized to GAPDH. SNHG14 expression was significantly downregulated in 83% (24/29) of the glioma tissues compared with that in the NATs (*p* < 0.001, Figure 1A). The relative expression levels of SNHG14 in 29 gliomas were also compared with those in 18 NBTs. SNHG14 expression was significantly lower in glioma tissues than that in NBTs (*p* < 0.001, Figure 1B). Furthermore, the expression levels of SNHG14 in the 29 tumor samples were stratified using three types of clinicopathological parameters (gender, age and WHO grade). However, no obvious significance was observed. The relative expression levels of SNHG14 in glioma cell lines were also measured. SNHG14 expression was significantly lower in glioma cell lines (U251 and U87) than that in normal HEB cells (Figure 1C). Collectively, the results showed that SNHG14 was downregulated in glioma.

### Overexpression of SNHG14 inhibits cell proliferation and cell invasion and promotes cell apoptosis in glioma

**Figure 2:**
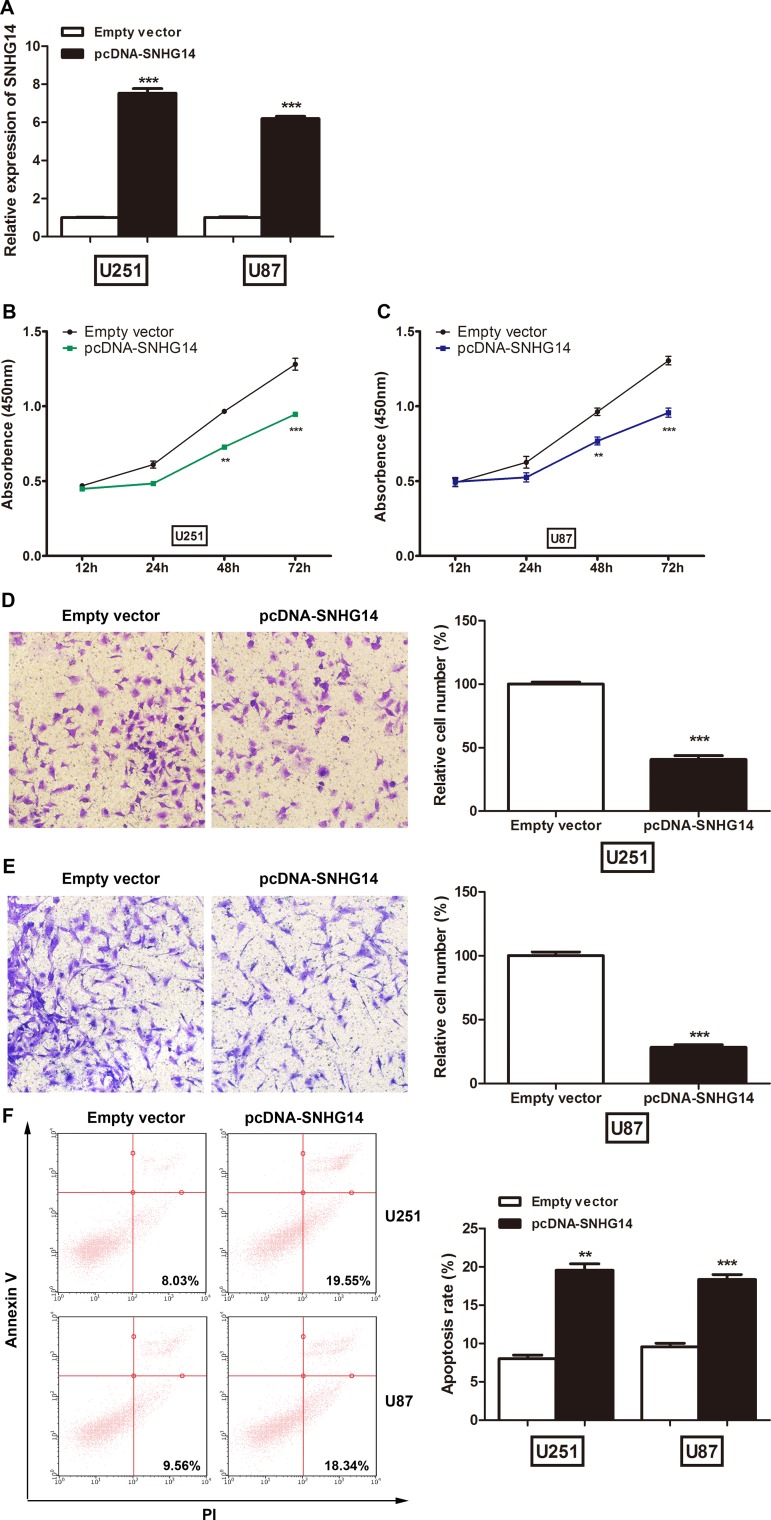
LncRNA SNHG14 suppressed glioma cell proliferation *in vitro* (**A**) The relative expression level of SNHG14 in glioma cell lines transfected with an empty vector or pcDNA-SNHG14. ^***^p < 0.001. (**B**) CCK-8 assays were used to determine glioma cell proliferation after U251 cells were transfected. ^**^p < 0.01, ^***^p < 0.001. (**C**) CCK-8 assays were used to determine glioma cell proliferation after U87 cells were transfected. ^**^p < 0.01, ^***^p < 0.001. (**D**) Cell invasion assays were used to determine glioma cell invasion U251 cells were transfected. ^***^p < 0.001. (**E**) Cell invasion assays were used to determine glioma cell invasion after U87 cells were transfected. ^***^p < 0.001. (**F**) Flow cytometry assays were used to determine apoptosis in U251 and U87 cells. ^**^p < 0.01, ^***^p < 0.001.

Because SNHG14 was downregulated in glioma, we evaluated the impact of SNHG14 overexpression in glioma cell lines to explore its biological functions. After transfection with pcDNA-SNHG14, SNHG14 expression was significantly increased by 7.5-fold and 6.2-fold in U251 and U87 cells, respectively (Figure 2A). The CCK-8 assay showed that SNHG14 overexpression significantly inhibited proliferation in U251 (Figure 2B) and U87 cells (Figure 2C). Cell invasion ability was determined by Transwell invasion assay. The number of invaded cells in the pcDNA-SNHG14-transfected group was significantly reduced when compared to that in the empty vector transfected group for both U251 (Figure 2D) and U87 cells (Figure 2E). Cell apoptosis was assessed by flow cytometry. The percentage of apoptotic cells was significantly increased after transfection with the SNHG1 plasmid in both U251 (from 7.7% to 19.9%) and U87 cells (from 9.6% to 19.3%) when compared with that of the negative control (Figure 2F).

### SNHG14 interacts with miR-92a-3p in glioma cells

**Figure 3:**
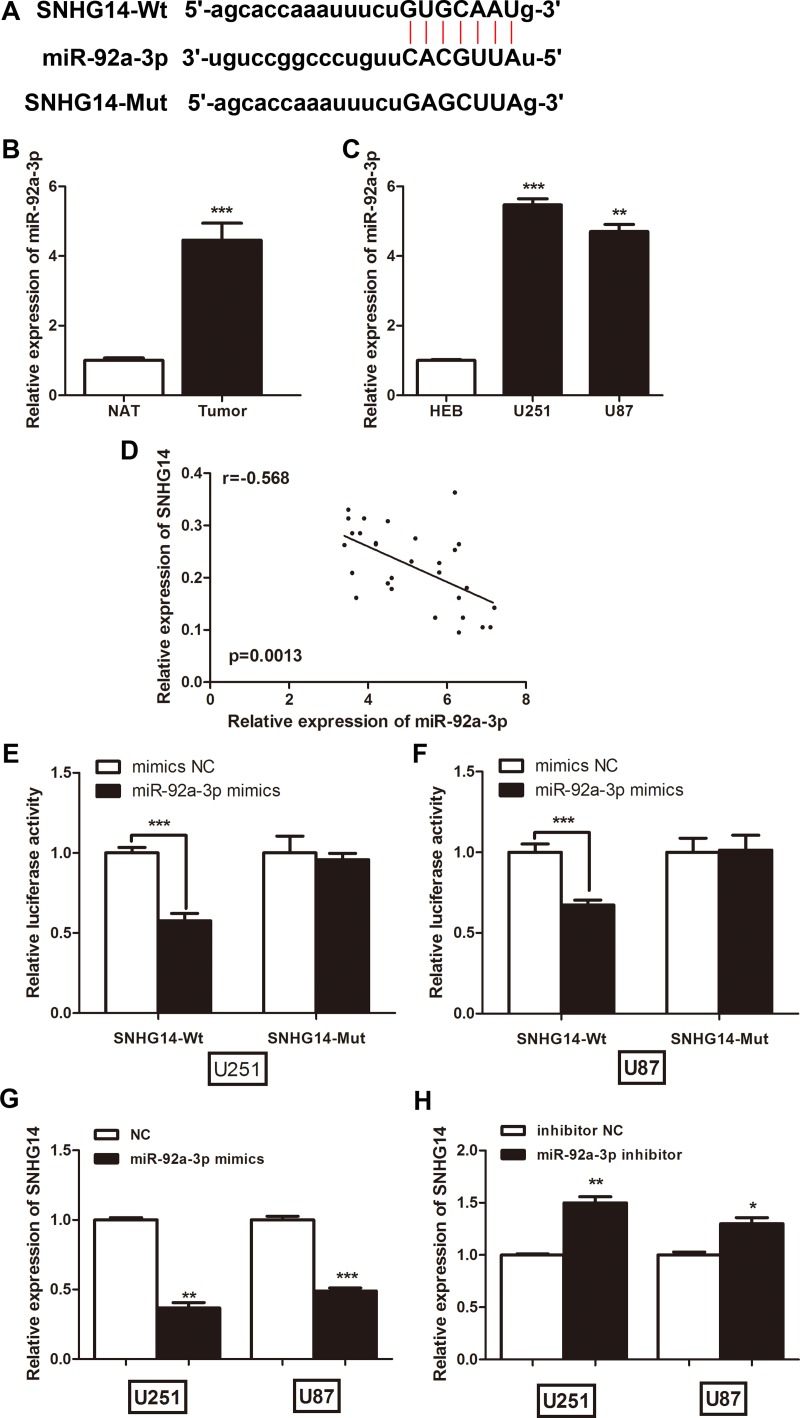
LncRNA SNHG14 directly interacted with miR-92a-3p in glioma cells (**A**) A bioinformatics tool (StarBase2.0) revealed the predicted binding sites between SNHG14 and miR-92a-3p. (**B**) The relative expression levels of miR-92a-3p in glioma tissues and NATs. ^***^p < 0.001. (**C**) The relative expression levels of miR-92a-3p in glioma cell lines (U251 and U87) and the HEB cell line. ^**^p <0.01, ^***^p < 0.001. (**D**) Pearson’s correlation analysis for the fold change of miR-92a-3p and SNHG14 in glioma tissues. (**E**) Luciferase reporter assays showed that miR-92a-3p overexpression significantly decreased the luciferase activity of SNHG14-Wt, but not SNHG14-Mut, in U251 cells. ^***^p < 0.001. (**F**) Luciferase reporter assays showed that miR-92a-3p overexpression significantly decreased the luciferase activity of SNHG14-Wt, but not SNHG14-Mut, in U87 cells. ^***^p < 0.001. (**G**) qRT-PCR analysis of SNHG14 expression levels in glioma cells treated with miR-92a-3p mimics. (**H**) qRT-PCR analysis of SNHG14 expression levels in glioma cells treated with miR-92a-3p inhibitor.

Accumulating evidence has suggested that miRNAs can interact with lncRNAs to regulate their expression levels and biological functions. The potential miRNA candidates targeting SNHG14 were predicted using StarBase2.0 [16]. The predicted sites of miR-92a-3p binding to the SNHG14 sequence are illustrated in Figure 3A. SNHG14 was downregulated in glioma tissues, whereas miR-92a-3p was significantly upregulated in the same paired 29 tumour and NAT samples (Figure 3B). miR-92a-3p expression was also upregulated in the glioma cell lines when compared with that in the normal cells (Figure 3C). A Spearman correlation analysis suggested a negative relationship between SNHG14 and miR-92a-3p expression (r = −0.568, *p* = 0.0013; Figure 3D). Subsequently, a luciferase reporter assay was performed to confirm whether miR-92a-3p could directly bind to SNHG14; cells were co-transfected with miR-92a-3p mimics and the SNHG14-Wt or SNHG14-Mut vector. The results revealed that miR-92a-3p significantly decreased the luciferase activity of SNHG14-Wt when compared with that of the negative control, but miR-92a-3p did not affect the luciferase activity of SNHG14-Mut in the U251 or U87 cell line (Figure 3E and 3F). We further clarified the regulatory relationship between SNHG14 and miR-92a-3p. miR-92a-3p significantly inhibited SNHG14 expression in both U251 and U87 cells (Figure 3G), whereas silencing SNHG14 did not affect miR-92a-3p expression (data not shown). Inversely, suppressing miR-92a-3p enhanced SNHG14 expression (Figure 3H). Taken together, these data indicated that miR-92a-3p could directly bind to SNHG14 in glioma cells.

### miR-92a-3p promotes glioma progression

**Figure 4:**
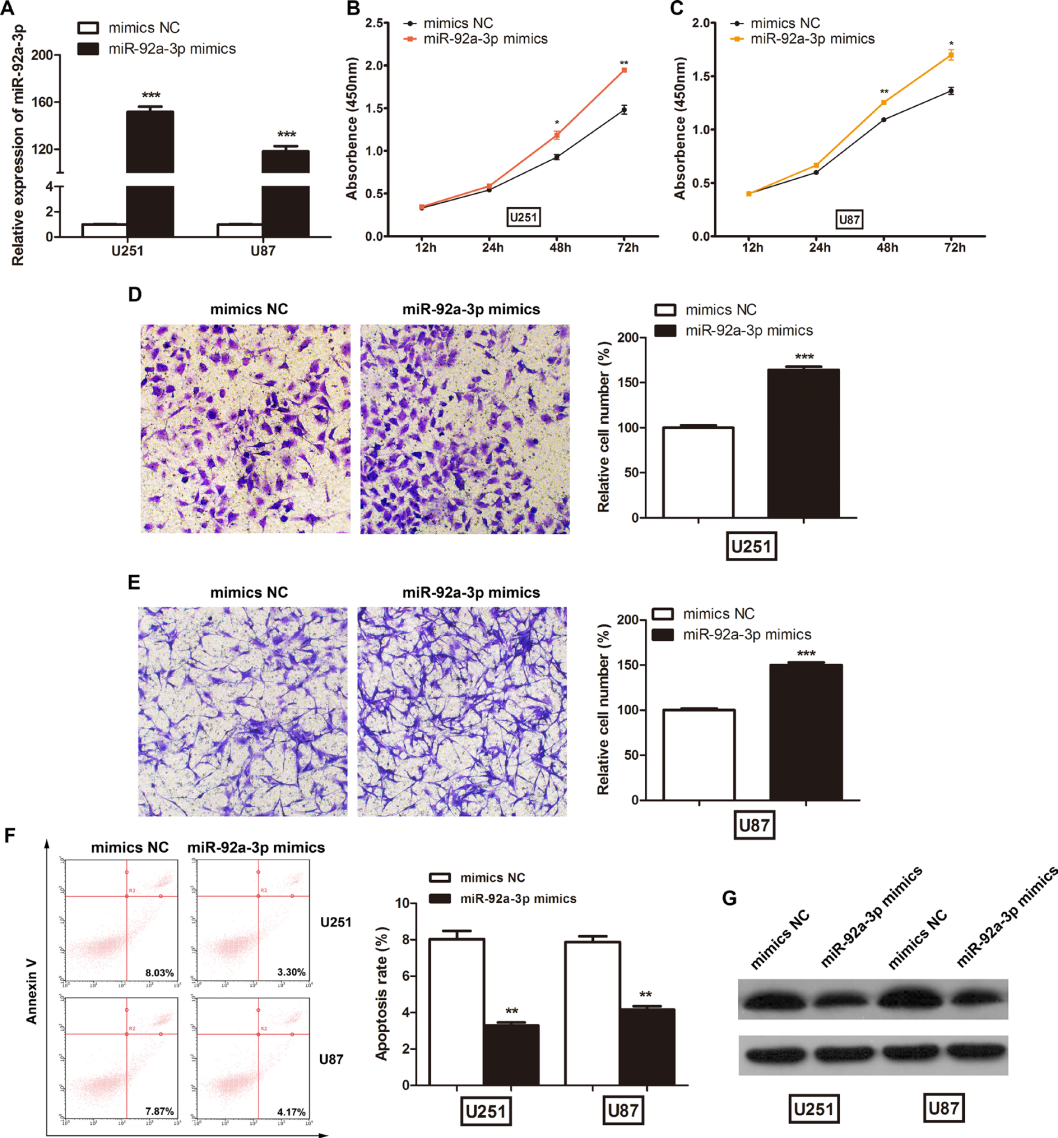
miR-92a-3p promoted glioma cell proliferation *in vitro* (**A**) The relative expression levels of miR-92a-3p in glioma cell lines transfected with miR-92a-3p mimics. ^***^p < 0.001. (**B**) CCK-8 assays were used to determine glioma cell proliferation after U251 cells were transfected with NC mimics or miR-92a-3p mimics. ^*^p < 0.05, ^**^p < 0.01. (**C**) CCK-8 assays were used to determine glioma cell proliferation after U87 cells were transfected with NC mimics or miR-92a-3p mimics. ^*^p < 0.05, ^**^p < 0.01. (**D**) Cell invasion assays were used to determine glioma cell invasion after U251 cells were transfected with miR-92a-3p mimics. ^***^p < 0.001. (**E**) Cell invasion assays were used to determine glioma cell invasion after U87 cells were transfected with miR-92a-3p mimics. ^***^p < 0.001. (**F**) Flow cytometry assays were used to determine apoptosis in U251 and U87 cells. ^**^p < 0.01. (**G**) The levels of Bim in U251 and U87 cells transfected with miR-92a-3p mimics determined by western blot.

The above results imply that miR-92a-3p may play an important role in glioma development; therefore, we investigated the biological functions of miR-92a-3p *in vitro*. We transfected NC mimics or miR-92a-3p mimics into glioma cells. After transfection, miR-92a-3p expression was significantly increased in the U251 and U87 cells (Figure 4A). The CCK-8 assay showed that miR-92a-3p overexpression significantly promoted cell proliferation in U251 (Figure 4B) and U87 cells (Figure 4C). Next, we performed cell invasion assays to assess the effect of miR-92a-3p on glioma cell invasion. The number of invaded cells was significantly increased after transfection with miR-92a-3p mimics compared with that after transfection with NC mimics in both U251 (Figure 4D) and U87 cells (Figure 4E). Furthermore, the percentage of apoptotic cells was slightly decreased after miR-92a-3p overexpression (Figure 4F). Though the apoptosis rate reduction was relatively small, it was statistically significant. miR-92a-3p has been reported to target and repress Bim expression in glioma [17]. The negative control mimics or miR-92a-3p mimics were transfected into glioma cells, and the levels of Bim protein were examined at 48 h after transfection. Western blot analysis revealed that the induced expression of miR-92a-3p significantly reduced Bim expression in both U251 and U87 cells, confirming that it is one of the direct targets of miR-92a-3p (Figure 4G).

### miR-92a-3p suppresses SNHG14 function

**Figure 5:**
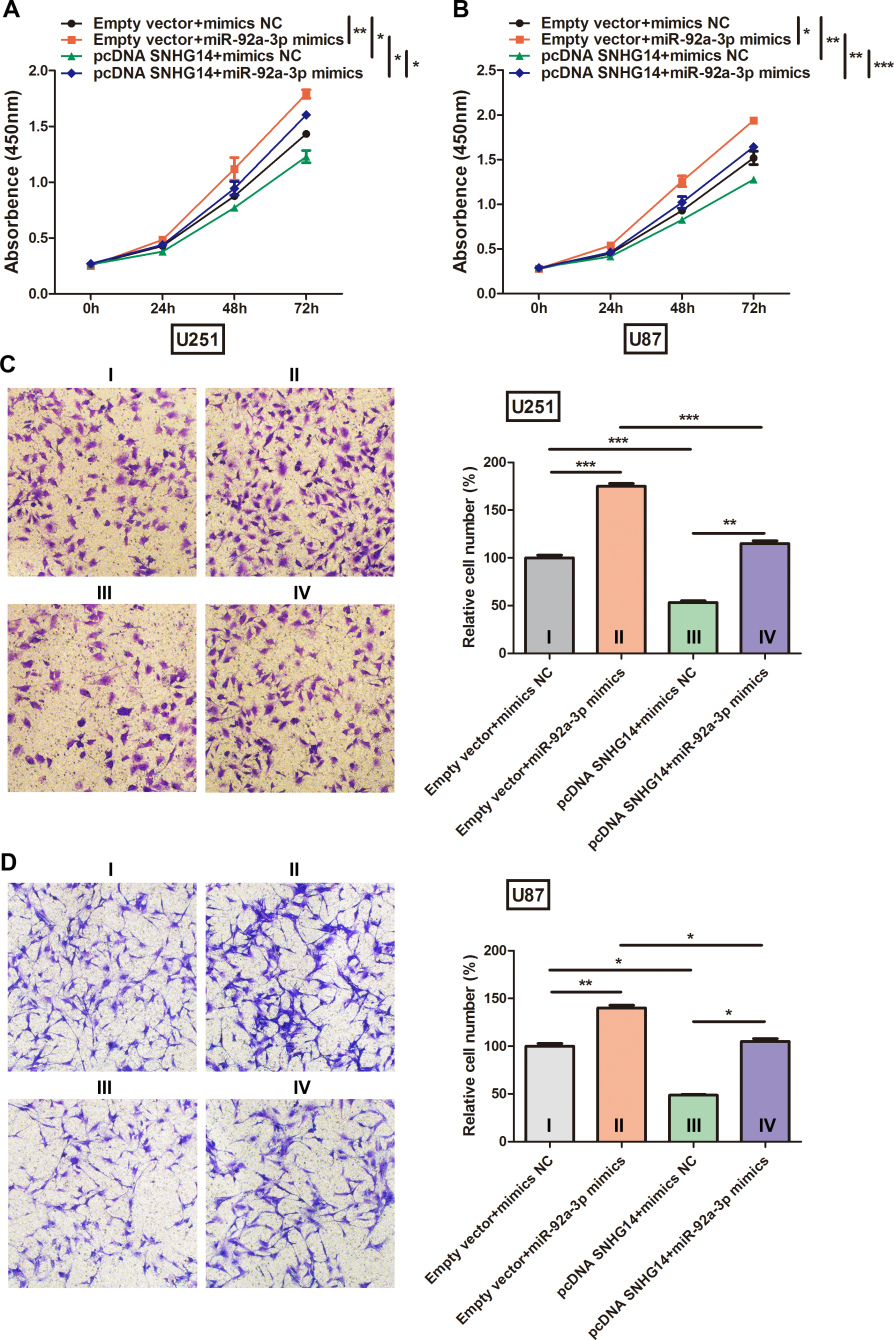

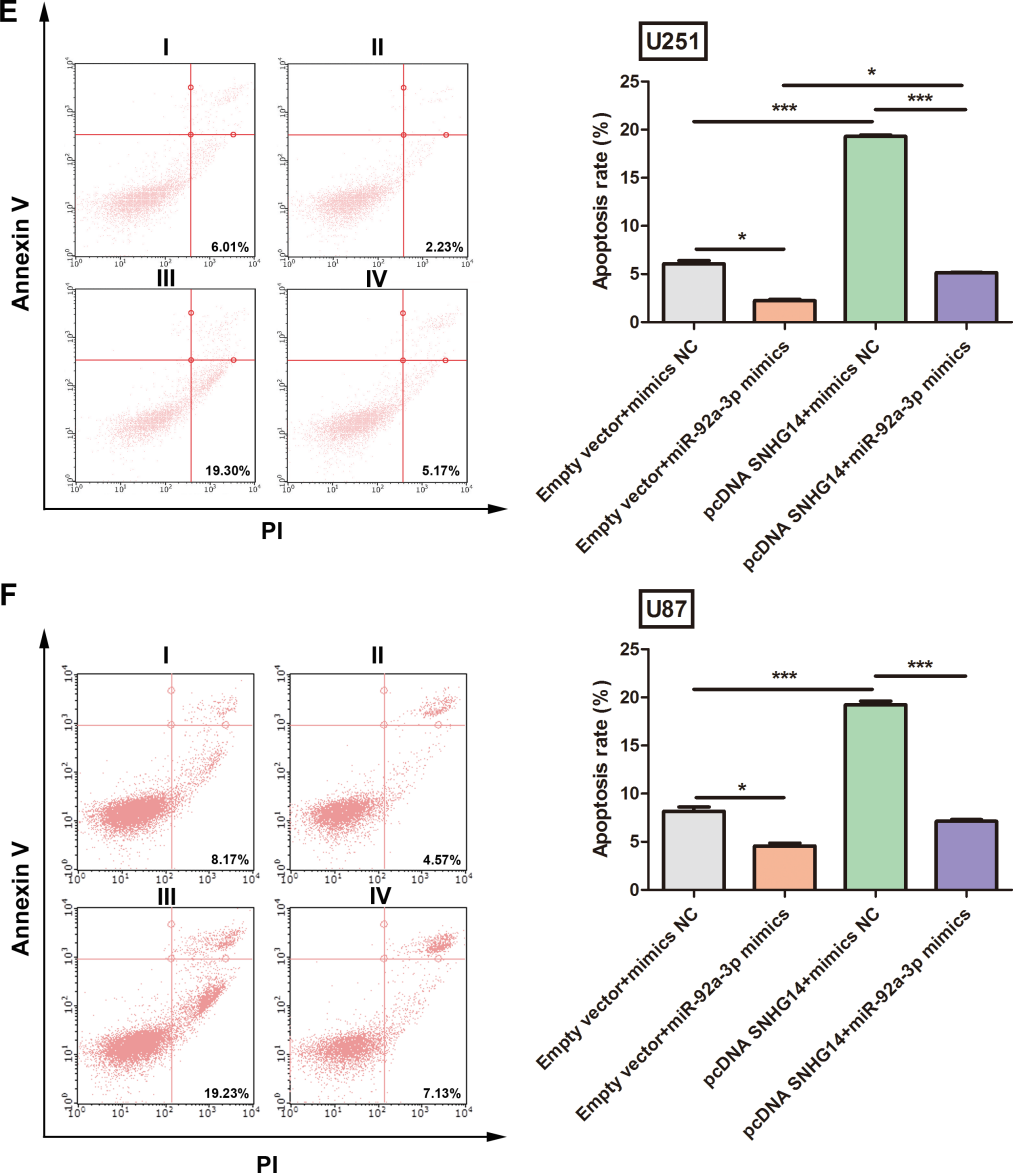
miR-92a-3p inhibited SNHG14 function in glioma (**A**) Cell viability was evaluated via CCK-8 assay after U251 cells were co-transfected with pcDNA-SNHG14 or miR-92a-3p mimics. (**B**) Cell viability was evaluated via CCK-8 assay after U87 cells were co-transfected with pcDNA-SNHG14 or miR-92a-3p mimics. (**C**) Cell invasion ability was evaluated via Transwell assay after U251 cells were co-transfected with pcDNA-SNHG14 or miR-92a-3p mimics. Left: Representative images from at least three independent experiments. Right: Statistical analysis of the cell invasion rate. (**D**) Cell invasion ability was evaluated via Transwell assay after U87 cells were co-transfected with pcDNA-SNHG14 or miR-92a-3p mimics. Left: Representative images from at least three independent experiments. Right: Statistical analysis of the cell invasion rate. (**E**) Apoptosis was evaluated by flow cytometry after U251 cells were co-transfected with pcDNA-SNHG14 or miR-92a-3p mimics. (**F**) Apoptosis was evaluated by flow cytometry after U87 cells were co-transfected with pcDNA-SNHG14 or miR-92a-3p mimics. Empty vector, pcDNA-SNHG14 negative control; mimics NC, miR-92a-3p mimics negative control.

To further investigate whether miR-92a-3p was involved in the effect of SNHG14 on glioma biological functions, U251 and U87 cells were transfected with miR-92a-3p mimics or pcDNA-SNHG14. The CCK-8 assay revealed that the miR-92a-3p mimics abrogated the effect of pcDNA-SNHG14 on reducing cell viability in U251 (Figure 5A) and U87 cells (Figure 5B). The number of invaded cells was significantly reduced after transfection with pcDNA-SNHG14, whereas miR-92a-3p mimics reversed this effect in both U251 (Figure 5C) and U87 cells (Figure 5D). Further, the percentage of apoptotic cells was significantly decreased by miR-92a-3p after SNHG14 overexpression in U251 (Figure 5E) and U87 cells (Figure 4F). These results suggested that SNHG14 was targeted by miR-92a-3p.

## DISCUSSION

Recent evidence of the role of non-coding RNAs in multiple cellular processes has improved our understanding of the biology of cancer [18]. Thus, the roles of dysregulated non-coding RNAs may provide new insights into the identification of potential therapeutic targets in clinical applications. In the present study, we identified that SNHG14 was significantly downregulated in glioma tissues and cell lines. Overexpression of SNHG14 markedly repressed cell proliferation and invasion and promoted cellular apoptosis in glioma cell lines. In addition, SNHG14 was experimentally confirmed as a direct and specific target of miR-92a-3p. miR-92a-3p was significantly upregulated in glioma and exerted oncogenic functions in glioma cells through inhibiting Bim. Moreover, miR-92a-3p overexpression significantly reversed the tumour suppression effects induced by SNHG14 overexpression in glioma. Taken together, SNHG14 inhibited cell proliferation and migration and promoted apoptosis by sponging miR-92a-3p in glioma.

Increasing evidence has demonstrated that lncRNAs are dysregulated in glioma and play essential roles in cancer development and progression. Some lncRNAs have been identified as oncogenic lncRNAs. MALAT1 promotes cell proliferation by sponging miR-101 [19] and increases cell resistance to temozolomide (TMZ) by regulating ZEB1 in glioma [20]. XIST exerts its oncogenic function by decreasing blood-tumour barrier permeability and promoting angiogenesis through the XIST-miR-137-Rac1 pathway [21] or the XIST-miR-137-FOXC1/ZO-2 pathway [22]. CCDC26 is upregulated in glioma and promotes the growth and metastasis of glioma by targeting miR-203 [23]. CCAT1 promotes glioma tumourigenesis by sponging miR-181b through its regulation of FGFR3 and PDGFRα [24]. KCNQ1OT1 promotes cell malignancy in the KCNQ1OT1-miR-370-CCNE2 axis pathway in glioma [25]. TP73-AS1 promotes brain glioma growth and invasion by sponging miR-142 to promote HMGB1 expression [26]. Some lncRNAs act as tumour suppressors in glioma. MEG3 suppresses glioma cell proliferation, migration and invasion by sponging miR-19a [27]. ENST00462717 suppresses proliferation, survival, and migration by partially regulating the MDM2/MAPK pathway in glioma [28]. PTCSC3 suppresses proliferation and induces apoptosis in U87 and U251 cells by targeting LRP6 to suppress the Wnt/β-catenin signalling pathway [29]. RP5-833A20.1 inhibits cell proliferation, metastasis and cell cycle progression by suppressing NFIA in glioma [30]. TUSC7 inhibits migration and promotes cellular apoptosis, largely bypassing miR-23b [31]. There are also some lncRNAs that play important roles in glioma stem cells. GAS5 suppresses the malignancy of human glioma stem cells via a miR-196a-5p/FOXO1 feedback loop [32]. NEAT1 maintains stem-like properties in glioma cells by modulating the miR-107/CDK6 pathway [33]. TALNEC2 increases the tumourigenic potential and the resistance to radiation in glioma stem cells [34]. As for SNHG14, Qi et al. reported that SNHG14 activated microglia cells in cerebral infarction by inhibition of miR-145-5p to increase PLA2G4A [11]. However, the activity of SNHG14 in glioma has not been previously described. In this study, we found that SNHG14 was downregulated in glioma cells and tissues, and its overexpression significantly inhibited cell proliferation and invasion, suggesting that SNHG14 acts as a tumour suppressor in glioma progression. As we know, each miRNA may repress up to hundreds of lncRNAs, while each lncRNA may be targeted by multiple miRNAs. For example, the lncRNA H19 was targeted by miR-29b-3p [35], miR-138-5p [36], miR-141-3p [37], miR-200b/c [38], miR-455-5p [39], and miR-675-5p [40]. Though up to date, SNHG14 is targeted by miR-145-5p and miR-92a-3p, it is very possible that more and more miRNAs may be identified to target SNHG14 in the future.

Accumulating evidence has shown that miRNAs play critical roles in tumour progression and development. miR-92a-3p, a member of the miR-17-92 cluster, is frequently dysregulated in a variety of cancer types and plays a critical role in cellular physiology [41]. miR-92a-3p promotes cell proliferation and cell cycle progression via inhibiting p21 [42] or FBXW7 [43] in cervical cancer. miR-92a-3p promotes epithelial-mesenchymal transition and regulates cell migration and invasion through the PI3K/AKT signalling pathway by targeting PTEN in non-small cell lung cancer [44]. miR-92a-3p increases the metastasis capability of nasopharyngeal carcinoma by targeting the PTEN/AKT pathway [45]. Inhibition of the function of miR-92a-3p represses the proliferation of pancreatic cancer cells through the miR-92a-3p/DUSP10/JNK signalling axis [46]. miR-92a-3p promotes tumour growth and reduces apoptosis by suppressing FBXW7 in osteosarcoma [47]. As in glioma, miR-92a-3p also functions as an onco-miRNA in tumour development. miR-92a-3p abrogation inhibits cell growth and induces apoptosis by targeting Bim [17]. miR-92a-3p promotes glioma cell malignancy *in vitro* and inhibits the stemness of glioma stem cells [48]. In this study, we found that miR-92a-3p could promote cell proliferation and invasion and reduce apoptosis by targeting Bim, which is consistent with previous reports. Unfortunately, there were no other reports about the lncRNAs targeted by miR-92a-3p in glioma. However, CDKN2B-AS was involved in the pathogenesis of coronary artery disease by targeting miR-92a-3p through GATA2, MAP1B and ARG1 regulation [49]. The TMZ is commonly used as the first-line therapy for glioma treatment, however, its resistance represents a major clinical challenge that leads to tumor relapse or progress. Emerging evidences have indicated that lncRNAs are correlated with glioma drug resistance, such as XIST [50], MALAT1 [20], CACSC2 [51], and H19 [52]. However, there were no reports about the relationship between miR-92a-3p/SNHG14 and TMZ resistance.

In summary, decreased expression of SNHG14 was observed in glioma patients. SNHG14 was targeted and inhibited by miR-92a-3p and acted as a tumour-suppressing gene, which inhibited the malignant behaviour of glioma cells. Our study facilitates the understanding of SNHG14 function in gliomagenesis and provides a novel therapeutic target.

## MATERIALS AND METHODS

### Clinical specimens

A total of 29 glioma tissues and paired normal adjacent tissues (NATs) were obtained from patients who accepted surgery at the Department of Neurosurgery, The First People’s Hospital of Changzhou. Eighteen samples of normal brain tissue (NBT) were collected from people who died in traffic accidents and had no prior pathologically detectable conditions. The patients consisted of 16 males and 13 females. Of these patients, 18 were older than 51 years of age, and 11 were younger. Of the 29 tumor samples, 2, 4, 9, and 14 samples were diagnosed as pilocytic astrocytoma (WHO grade I), diffuse astrocytoma (WHO grade II), anaplastic astrocytoma (WHO grade III), or glioblastoma multiforme (WHO grade IV), respectively. The study was approved by the Research Ethics Board of The First People’s Hospital of Changzhou. Biopsy specimens were immediately snap frozen in liquid nitrogen and stored at –80°C until further processing. None of the patients were treated with radiotherapy or chemotherapy before surgery.

### Cell culture and transfection

The human glioma cell lines U251 and U87 were purchased from the Chinese Academy of Science Cell Bank (Shanghai, China). The normal brain glial cell line HEB was purchased from Beierbo Company (Nanjing, China). These cell lines were maintained in DMEM (Invitrogen, USA) supplemented with 10% foetal bovine serum (Invitrogen, USA) and cultured in a humidified incubator at 37°C and 5% CO_2_. The plasmid for lncRNA-SNHG14, named pcDNA-SNHG14, was constructed by introducing the cDNA sequence of SNHG14 into the pcDNA3.1 expression vector (Invitrogen, USA). The miR-92a-3p mimics, negative control mimics (NC mimics), miR-92a-3p inhibitor, and negative control inhibitor (NC inhibitor) were purchased from GenePharma (Shanghai, China). Transfection was performed by using Lipofectamine 2000 (Invitrogen, USA) according to the manufacturer’s instructions.

### Quantitative real-time PCR (qRT-PCR) analysis

Total RNA was extracted from fresh frozen tissues or cultured cells using Trizol reagent (Life Technologies, USA). The ratio of the absorbance at 260 and 280 nm (A_260/280_) measured with a Beckman DU800 spectrophotometer was used to assess the purity of the nucleic acids. The expression of SNHG14 was determined using a method similar to our previous work [53]. First-strand cDNA was synthesized by using Reverse Transcriptase SuperScript III (Invitrogen, USA) and approximately 2 μg of total RNA. The expression levels of SNHG14 in glioma tissues or cultured cells were quantified using an ABI Prism 7500 system (Applied Biosystems, USA). GAPDH was chosen as the internal standard. The expression levels of mature miR-92a-3p were quantified using TaqMan miRNA probes and an ABI Prism 7500 system (Applied Biosystems, USA) according to previous methods [54]. The relative levels of miR-92a-3p in cultured cells or tissue samples were normalized to those of U6. All reactions were performed in triplicate.

### Cell proliferation assay

Cells were treated using different transfection conditions and plated in 96-well plates. Cell proliferation was assessed daily for three consecutive days using CCK-8 assay kits (Beyotime, China) according to the manufacturer’s protocol. Cell viability was determined by measuring the absorbance at a wavelength of 450 nm on an Elx800 system (BioTek, USA). All of the experiments were performed in sextuplicate and repeated at least three times.

### Cell invasion assay

Cell invasion assays were performed using Matrigel-coated Transwell plates (Biosciences, USA) as previously reported [55]. Cells were harvested 24 h after transfection and were seeded into the upper chamber with serum-free medium. Fresh medium containing 10% serum was added to the lower chamber to act as the chemoattractant. After 48 h of incubation, the invaded cells on the bottom surface were fixed and stained with 0.1% crystal violet, and the non-invaded cells were removed with a cotton swab. Six random fields in each well were counted under an IX71 microscope (Olympus, Japan) with Image-Pro Insight software (Olympus, Japan). The mean number of invading cells was expressed as a percentage relative to the control. The data are represented as the mean ± SD from at least three independent experiments.

### Cell apoptosis assay

Cell apoptosis was analysed by flow cytometry on a Guava EasyCyte 6HT-2L flow cytometer (Merck Millipore, Germany) as previously described [53]. The transfected cells were harvested and dual-stained with propidium iodide (PI) and Annexin V-FITC using an apoptosis detection kit (Beyotime, China). The results were analysed using Guavasoft 2.7 software (Merck Millipore, Germany).

### Luciferase reporter assay

A SNHG14 fragment containing the predicted miR-92a-3p binding site and its mutant sequence were cloned into a pGL3 vector (Promega, USA). The generated vectors were sequenced and named SNHG14-Wt and SNHG14-Mut. Cells were seeded in 96-well plates at 1 × 10^4^ cells per well and transfected with different treatments using Lipofectamine 2000 to 75% confluence. At 48 h after transfection, the relative luciferase activities were measured by using a dual-luciferase reporter assay system (Promega, USA) according to the manufacturer’s instructions.

### Western blot

Western blot assays were performed according to our previous method [54]. Primary antibodies against Bim (Abcam, USA), β-actin (Cell Signaling Technology, USA) and IgG-HRP (Sigma, USA) were used. Protein levels were detected on a Bio-Rad ChemiDocXRS system (Bio-Rad, USA) and quantified using Quantity One software (Bio-Rad, USA).

### Statistical analysis

Data were analysed with GraphPad Prism 5.0 (GraphPad Software, USA). All values are presented as the mean ± SD. Student’s *t*-test was used for comparisons between two groups, and one-way ANOVA was used for multi-group comparisons. Pearson’s correlation coefficients were calculated to determine the significance of the relationship between SNHG14 and miR-92a-3p expression. Differences were considered statistically significant when *p* < 0.05. All the results were from at least three independent experiments.

## Abbreviations

lncRNA: long non-coding RNA
GBM: glioblastoma
SNHG14: small nucleolar RNA host gene 14
miRNA: microRNA
3’-UTR: 3’-untranslated region
TMZ: temozolomide

## References

[1] Ricard D, Idbaih A, Ducray F, Lahutte M, Hoang-Xuan K, Delattre JY (2012). Primary brain tumours in adults. Lancet.

[2] Gladson CL, Prayson RA, Liu WM (2010). The pathobiology of glioma tumors. Annu Rev Pathol.

[3] Khan UA, Bhavsar A, Asif H, Karabatsou K, Leggate JR, Sofat A, Kamaly-Asl ID (2015). Treatment by specialist surgical neurooncologists improves survival times for patients with malignant glioma. J Neurosurg.

[4] Ponting CP, Oliver PL, Reik W (2009). Evolution and functions of long noncoding RNAs. Cell.

[5] Qi P, Du X (2013). The long non-coding RNAs, a new cancer diagnostic and therapeutic gold mine. Mod Pathol.

[6] Wapinski O, Chang HY (2011). Long noncoding RNAs and human disease. Trends Cell Biol.

[7] Tsai MC, Spitale RC, Chang HY (2011). Long intergenic noncoding RNAs: new links in cancer progression. Cancer Res.

[8] Ramos AD, Attenello FJ, Lim DA (2016). Uncovering the roles of long noncoding RNAs in neural development and glioma progression. Neurosci Lett.

[9] Wang L, Yu Z, Sun S, Peng J, Xiao R, Chen S, Zuo X, Cheng Q, Xia Y (2017). Long non-coding RNAs: potential molecular biomarkers for gliomas diagnosis and prognosis. Rev Neurosci.

[10] Sadikovic B, Fernandes P, Zhang VW, Ward PA, Miloslavskaya I, Rhead W, Rosenbaum R, Gin R, Roa B, Fang P (2014). Mutation Update for UBE3A variants in Angelman syndrome. Hum Mutat.

[11] Qi X, Shao M, Sun H, Shen Y, Meng D, Huo W (2017). Long non-coding RNA SNHG14 promotes microglia activation by regulating miR-145-5p/PLA2G4A in cerebral infarction. Neuroscience.

[12] He L, Hannon GJ (2004). MicroRNAs: small RNAs with a big role in gene regulation. Nat Rev Genet.

[13] Bushati N, Cohen SM (2007). microRNA functions. Annu Rev Cell Dev Biol.

[14] Ryan BM, Robles AI, Harris CC (2010). Genetic variation in microRNA networks: the implications for cancer research. Nat Rev Cancer.

[15] Adams BD, Parsons C, Walker L, Zhang WC, Slack FJ (2017). Targeting noncoding RNAs in disease. J Clin Invest.

[16] Li JH, Liu S, Zhou H, Qu LH, Yang JH (2014). starBase v2.0: decoding miRNA-ceRNA, miRNA-ncRNA and protein-RNA interaction networks from large-scale CLIP-Seq data. Nucleic Acids Res.

[17] Niu H, Wang K, Zhang A, Yang S, Song Z, Wang W, Qian C, Li X, Zhu Y, Wang Y (2012). miR-92a is a critical regulator of the apoptosis pathway in glioblastoma with inverse expression of BCL2L11. Oncol Rep.

[18] Bhan A, Soleimani M, Mandal SS (2017). Long Noncoding RNA and Cancer: A New Paradigm. Cancer Res.

[19] Li Z, Xu C, Ding B, Gao M, Wei X, Ji N (2017). Long non-coding RNA MALAT1 promotes proliferation and suppresses apoptosis of glioma cells through derepressing Rap1B by sponging miR-101. J Neurooncol.

[20] Li H, Yuan X, Yan D, Li D, Guan F, Dong Y, Wang H, Liu X, Yang B (2017). Long Non-Coding RNA MALAT1 Decreases the Sensitivity of Resistant Glioblastoma Cell Lines to Temozolomide. Cell Physiol Biochem.

[21] Wang Z, Yuan J, Li L, Yang Y, Xu X, Wang Y (2017). Long non-coding RNA XIST exerts oncogenic functions in human glioma by targeting miR-137. Am J Transl Res.

[22] Yu H, Xue Y, Wang P, Liu X, Ma J, Zheng J, Li Z, Li Z, Cai H, Liu Y (2017). Knockdown of long non-coding RNA XIST increases blood-tumor barrier permeability and inhibits glioma angiogenesis by targeting miR-137. Oncogenesis.

[23] Wang S, Hui Y, Li X, Jia Q (2017). Silencing of lncRNA-CCDC26 Restrains the Growth and Migration of Glioma Cells *In Vitro* and *In Vivo* Via Targeting miR-203. Oncol Res.

[24] Cui B, Li B, Liu Q, Cui Y (2017). lncRNA CCAT1 Promotes Glioma Tumorigenesis by Sponging miR-181b. J Cell Biochem.

[25] Gong W, Zheng J, Liu X, Liu Y, Guo J, Gao Y, Tao W, Chen J, Li Z, Ma J, Xue Y (2017). Knockdown of Long Non-Coding RNA KCNQ1OT1 Restrained Glioma Cells’ Malignancy by Activating miR-370/CCNE2 Axis. Front Cell Neurosci.

[26] Zhang R, Jin H, Lou F (2017). The long non-coding RNA TP73-AS1 interacted with miR-142 to modulate brain glioma growth through HMGB1/RAGE pathway. J Cell Biochem.

[27] Qin N, Tong GF, Sun LW, Xu XL (2017). Long Noncoding RNA MEG3 Suppresses Glioma Cell Proliferation, Migration, and Invasion by Acting as a Competing Endogenous RNA of miR-19a. Oncol Res.

[28] Wang A, Meng M, Zhao X, Kong L (2017). Long non-coding RNA ENST00462717 suppresses the proliferation, survival, and migration by inhibiting MDM2/MAPK pathway in glioma. Biochem Biophys Res Commun.

[29] Xia S, Ji R, Zhan W (2017). Long noncoding RNA papillary thyroid carcinoma susceptibility candidate 3 (PTCSC3) inhibits proliferation and invasion of glioma cells by suppressing the Wnt/beta-catenin signaling pathway. BMC Neurol.

[30] Kang CM, Hu YW, Nie Y, Zhao JY, Li SF, Chu S, Li HX, Huang QS, Qiu YR (2016). Long non-coding RNA RP5–833A20.1 inhibits proliferation, metastasis and cell cycle progression by suppressing the expression of NFIA in U251 cells. Mol Med Rep.

[31] Shang C, Guo Y, Hong Y, Xue YX (2016). Long Non-coding RNA TUSC7, a Target of miR-23b, Plays Tumor-Suppressing Roles in Human Gliomas. Front Cell Neurosci.

[32] Zhao X, Liu Y, Zheng J, Liu X, Chen J, Liu L, Wang P, Xue Y (2017). GAS5 suppresses malignancy of human glioma stem cells via a miR-196a-5p/FOXO1 feedback loop. Biochim Biophys Acta.

[33] Yang X, Xiao Z, Du X, Huang L, Du G (2017). Silencing of the long non-coding RNA NEAT1 suppresses glioma stem-like properties through modulation of the miR-107/CDK6 pathway. Oncol Rep.

[34] Brodie S, Lee HK, Jiang W, Cazacu S, Xiang C, Poisson LM, Datta I, Kalkanis S, Ginsberg D, Brodie C (2017). The novel long non-coding RNA TALNEC2, regulates tumor cell growth and the stemness and radiation response of glioma stem cells. Oncotarget.

[35] Lu YF, Liu Y, Fu WM, Xu J, Wang B, Sun YX, Wu TY, Xu LL, Chan KM, Zhang JF, Li G (2017). Long noncoding RNA H19 accelerates tenogenic differentiation and promotes tendon healing through targeting miR-29b-3p and activating TGF-beta1 signaling. FASEB J.

[36] Yang Q, Wang X, Tang C, Chen X, He J (2017). H19 promotes the migration and invasion of colon cancer by sponging miR-138 to upregulate the expression of HMGA1. Int J Oncol.

[37] Zhou X, Ye F, Yin C, Zhuang Y, Yue G, Zhang G (2015). The Interaction Between MiR-141 and lncRNA-H19 in Regulating Cell Proliferation and Migration in Gastric Cancer. Cell Physiol Biochem.

[38] Zhou W, Ye XL, Xu J, Cao MG, Fang ZY, Li LY, Guan GH, Liu Q, Qian YH, Xie D (2017). The lncRNA H19 mediates breast cancer cell plasticity during EMT and MET plasticity by differentially sponging miR-200b/c and let-7b. Sci Signal.

[39] Huang ZW, Tian LH, Yang B, Guo RM (2017). Long Noncoding RNA H19 Acts as a Competing Endogenous RNA to Mediate CTGF Expression by Sponging miR-455 in Cardiac Fibrosis. DNA Cell Biol.

[40] Chen S, Bu D, Ma Y, Zhu J, Chen G, Sun L, Zuo S, Li T, Pan Y, Wang X, Liu Y, Wang P (2017). H19 Overexpression Induces Resistance to 1,25(OH)2D3 by Targeting VDR Through miR-675-5p in Colon Cancer Cells. Neoplasia.

[41] Li M, Guan X, Sun Y, Mi J, Shu X, Liu F, Li C (2014). miR-92a family and their target genes in tumorigenesis and metastasis. Exp Cell Res.

[42] Su Z, Yang H, Zhao M, Wang Y, Deng G, Chen R (2017). MicroRNA-92a Promotes Cell Proliferation in Cervical Cancer via Inhibiting p21 Expression and Promoting Cell Cycle Progression. Oncol Res.

[43] Zhou C, Shen L, Mao L, Wang B, Li Y, Yu H (2015). miR-92a is upregulated in cervical cancer and promotes cell proliferation and invasion by targeting FBXW7. Biochem Biophys Res Commun.

[44] Lu C, Shan Z, Hong J, Yang L (2017). MicroRNA-92a promotes epithelial-mesenchymal transition through activation of PTEN/PI3K/AKT signaling pathway in non-small cell lung cancer metastasis. Int J Oncol.

[45] Zhang H, Cao H, Xu D, Zhu K (2016). MicroRNA-92a promotes metastasis of nasopharyngeal carcinoma by targeting the PTEN/AKT pathway. Onco Targets Ther.

[46] He G, Zhang L, Li Q, Yang L (2014). miR-92a/DUSP10/JNK signalling axis promotes human pancreatic cancer cells proliferation. Biomed Pharmacother.

[47] Jiang X, Li X, Wu F, Gao H, Wang G, Zheng H, Wang H, Li J, Chen C (2017). Overexpression of miR-92a promotes the tumor growth of osteosarcoma by suppressing F-box and WD repeat-containing protein 7. Gene.

[48] Song H, Zhang Y, Liu N, Zhao S, Kong Y, Yuan L (2016). miR-92a-3p Exerts Various Effects in Glioma and Glioma Stem-Like Cells Specifically Targeting CDH1/beta-Catenin and Notch-1/Akt Signaling Pathways. Int J Mol Sci.

[49] Cheng M, An S, Li J (2017). CDKN2B-AS may indirectly regulate coronary artery disease-associated genes via targeting miR-92a. Gene.

[50] Du P, Zhao H, Peng R, Liu Q, Yuan J, Peng G, Liao Y (2017). LncRNA-XIST interacts with miR-29c to modulate the chemoresistance of glioma cell to TMZ through DNA mismatch repair (MMR) pathway. Biosci Rep.

[51] Liao Y, Shen L, Zhao H, Liu Q, Fu J, Guo Y, Peng R, Cheng L (2017). LncRNA CASC2 Interacts With miR-181a to Modulate Glioma Growth and Resistance to TMZ Through PTEN Pathway. J Cell Biochem.

[52] Jiang P, Wang P, Sun X, Yuan Z, Zhan R, Ma X, Li W (2016). Knockdown of long noncoding RNA H19 sensitizes human glioma cells to temozolomide therapy. Onco Targets Ther.

[53] Wang Q, Li Q, Zhou P, Deng D, Xue L, Shao N, Peng Y, Zhi F (2017). Upregulation of the long non-coding RNA SNHG1 predicts poor prognosis, promotes cell proliferation and invasion, and reduces apoptosis in glioma. Biomed Pharmacother.

[54] Deng D, Wang L, Chen Y, Li B, Xue L, Shao N, Wang Q, Xia X, Yang Y, Zhi F (2016). MicroRNA-124-3p regulates cell proliferation, invasion, apoptosis, and bioenergetics by targeting PIM1 in astrocytoma. Cancer Sci.

[55] Deng D, Xue L, Shao N, Qu H, Wang Q, Wang S, Xia X, Yang Y, Zhi F (2016). miR-137 acts as a tumor suppressor in astrocytoma by targeting RASGRF1. Tumour Biol.

